# The Causal Relationship between PCSK9 Inhibitors and Osteoporosis Based on Drug-Targeted Mendelian Combined Mediation Analysis

**DOI:** 10.1007/s00223-024-01228-x

**Published:** 2024-05-24

**Authors:** Naidan Zhang, Chaixia Ji, Li Liu, Ermei Ye, Chengliang Yuan

**Affiliations:** 1 Department of Laboratory Medicine, Peoples Hospital of Deyang City, No 173, the First Section of North Taishan Road, Deyang, 618000 China; 2 Department of Medical Technology, Chengdu University of Traditional Chinese Medicine, Chengdu, 610075 China

**Keywords:** PCSK9 inhibitors, Osteoporosis, Drug-targeted mendelian randomization, Mediation analysis

## Abstract

PCSK9 inhibitors have been shown to lower serum low density lipoprotein cholesterol (LDL*-*C) levels and are considered integral in the treatment of cardiovascular diseases. However, the potential association between PCSK9 inhibitors and osteoporosis is unclear now. In this study, drug-targeted mendelian randomization (MR) was utilized in conjunction with mediation analysis including bone mineral density (BMD), total 25-hydroxyvitamin D (T25(OH)D) levels and calcium supplementation to investigate the causal relationship between PCSK9 inhibitors and osteoporosis. The LDL-C level was chosen as the exposure variable in a sample size of 173,082 individuals. We conducted a MR analysis on the relationship between PCSK9 inhibitors and osteoporosis, elucidating the mediators involved. Utilizing the inverse variance weighted (IVW*)* method, we found the risk of osteoporosis was reduced by 0.6% in those who used PCSK9 inhibitors compared with non-users (OR: 0.994, 95%*CI*: 0.991–0.998, *P* < 0.001). In people aged 30–45 years, the risk of low BMD was 1.176 times higher among PCSK9 inhibitor users compared to non-users (OR: 1.176, 95%*CI*: 1.017–1.336, *P* = 0.045). Conversely, people aged 45–60 years who used PCSK9 inhibitors had a 14.9% lower risk of low BMD compared to non-users (OR: 0.851, 95%*CI*: 0.732–0.968, *P* = 0.007). Mediation analysis revealed that 43.33% of the impact of PCSK9 inhibitors on osteoporosis was mediated through BMD levels, with the remaining 56.67% being a direct effect. Effects of PCSK9 inhibitors on BMD levels varied in different ages. In addition, the risk of high serum T25(OH)D levels were 1.091 times among PCSK9 inhibitor users compared to non-users (OR: 1.091, 95%*CI*: 1.065–1.112,* P* < 0.001), providing valuable insights for clinicians.

**Supplementary Information:**

The online version contains supplementary material available at 10.1007/s00223-024-01228-x.

## Introduction

Osteoporosis is a systemic metabolic bone disease. It is characterized by bone loss, deterioration of bone tissue microarchitecture, and an increased risk of fragility fractures [1]. Patients with osteoporosis often present with metabolic diseases, such as dyslipidemia and abdominal obesity [2]. Dyslipidemia is characterized by elevated total cholesterol, triglycerides, low density lipoprotein-C (LDL-C), and decreased of high-density lipoprotein-C (HDL-C) levels [3]. A clinical study found a negative correlation between LDL-C and lumbar bone density in individuals aged 20–59 years [4]. Furthermore, an in vitro study demonstrated that LDL adsorption negatively regulated osteoblast behaviors, such as adhesion, proliferation and differentiation [5]. These studies suggest the need for further research into the relationship between LDL-C and osteoporosis.

Proprotein convertase subtilisin/kexin type 9 (PCSK9) is a serine protease. It binds to low-density lipoprotein (LDL) receptors, leading to intracellular degradation and reduced serum LDL clearance [6]. Compared to statins, the use of PCSK9 inhibitors not only lowers LDL levels but also demonstrates a superior short-term safety profile [7]. Recent studies suggest that PCSK9 inhibitors have pleiotropic effects beyond lipid regulation, potentially playing an important role in anti-inflammatory responses and immune cell regulation [8]. Macrophage polarization may influence osteoblast differentiation, promoting bone formation and mineralization [9]. However, activated macrophages can also release proinflammatory cytokines that induce bone loss through osteoclastogenesis and bone resorption [10]. Although drugs targeting LDL-C are common in clinical practice, further research is needed to understand the impact of PCSK9 inhibitors on osteoporosis.

Drug-targeted mendelian randomization (MR) is an algorithm that utilizes drug-related factors as instrumental variables (IVs) to assess the causal relationship between a drug and a disease by screening IVs near the drug gene [11]. Mediation analysis, as an essential extension of MR, helps confirm causal relationships between exposure and outcome factors. By integrating drug-targeted MR with mediation analysis, we aim to clarify the causal relationship between PCSK9 inhibitors and osteoporosis, offering valuable insights for clinicians in treatment decision-making.

## Materials and Methods

### Research Process and IVs Selection

Based on the clinical study that PCSK9 inhibitors can effectively reduce LDL-C levels, our research delved into the potential relationship between PCSK9 inhibitors and osteoporosis. We selected the LDL-C dataset (ieu-a-300) from the GWAS catalog (https://gwas.mrcieu.ac.uk/) as exposure factor. This dataset included a total of 173,082 individuals with 2,437,752 single nucleotide polymorphisms (SNPs). The dataset comprised 37 studies predominantly involving European individuals, with additional studies focusing on East Asian, South Asian, and African populations [12]. Samples for LDL-C analysis were collected from fasting individuals, with those taking lipid-lowering medication being excluded. Criteria for IVs selection included screening SNPs associated with LDL-C from the dataset (ieu-a-300), choosing SNPs meeting genome-wide significance (*P* < 5 × 10^–8^), and ensuring no significant linkage disequilibrium (*r*^*2*^ < 0.3). A total of 403 SNPs were initially identified, followed by further selection of 13 SNPs located within ± 100 clumping distance (kb) of the PCSK9 gene as IVs (Supplementary Table 1).

In this study, we utilized a dataset of coronary heart disease (CHD) as the positive control for drug-targeted MR analysis. The case status of CHD was defined using a comprehensive coronary artery disease (CAD) diagnosis, which encompassed acute coronary syndrome, coronary stenosis > 50%, chronic stable angina, or myocardial infarction (ieu-a-7). Utilizing CHD as the positive control not only ensured the reliability of the experimental outcomes but also highlighted the potential clinical implications of the drug-targeted MR analysis results. The dataset included a total of 184,305 individuals (case = 60,801, control = 123,504) and 9,455,779 SNPs. The initial focus was on assessing the causal relationship between PCSK9 inhibitors and CHD, with a *P*_IVW_ < 0.05 indicating CHD as a positive control.

Following the verification of the causal relationship between PCSK9 inhibitors and CHD, we selected the osteoporosis dataset as the outcome factor (ukb-b-12141). This dataset comprised 462,933 individuals (case = 7,547, control = 455,386) with 9,851,867 SNPs. Osteoporosis diagnosis was established through a bone dual energy X-ray examination, with a T-score below -2.5 indicating the presence of osteoporosis [13]. Exclusion criteria involved the presence of bone destruction from neoplastic disease in all patients. The PHESANT (PHEnome Scan ANalysis Tool) was used to obtain the dataset from the UK Biobank [14]. To mitigate strong heterogeneity, all palindromic SNPs among the 13 IVs were removed using the “Two Sample MR” package. Subsequently, a “two sample MR” analysis was conducted, combining the IVs to investigate the relationship between PCSK9 inhibitors and osteoporosis. Heterogeneity, pleiotropy, sensitivity and bias were calculated.

**Fig. 1:**
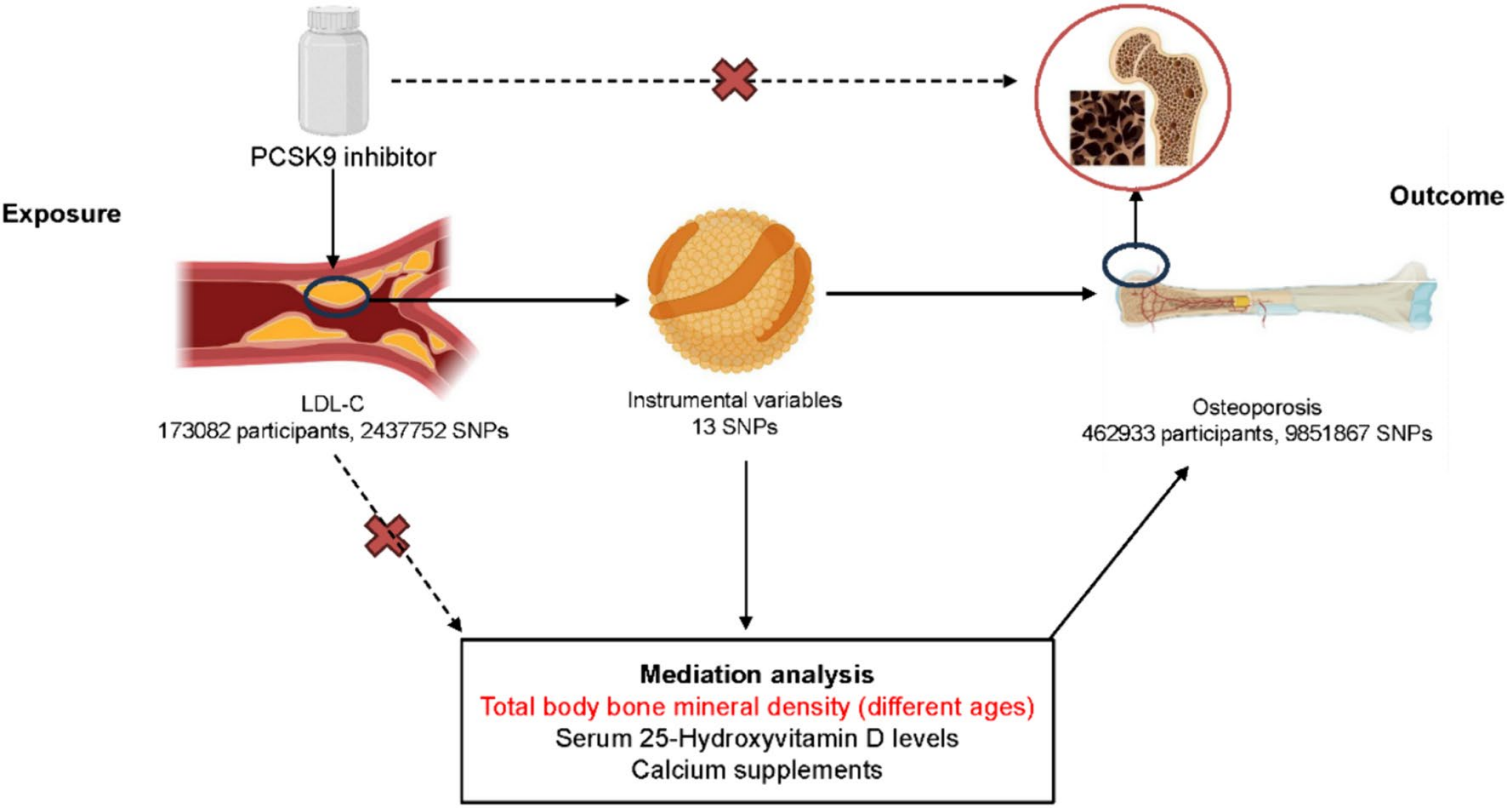
Study flow chart. This study was devided into MR analysis and mediation analysis. MR analysis was used to assess whether LDL-C level based on PCSK9 inhibitors had a causal relationship with osteoporosis. Mediation analysis was used to assess whether mediation effect was existing. MR, Mendelian randomization; SNPs, single nucleotide polymorphisms

Drug-targeted MR analysis was utilized to investigate the potential causal relationship between PCSK9 inhibitors and osteoporosis. This method was advantageous as it was not influenced by confounding factors, allowing for a more accurate exploration of mediation effects. To further examine the mediation effect, the “two step MR” method was employed, focusing on common indicators such as bone mineral density (BMD) levels, total 25-hydroxyvitamin D (T25(OH)D) levels, and calcium supplementations (Fig. 1).

### Causal Effect and Heterogeneity

In this study, four algorithms (MR Egger, weighted median, inverse variance weighted and weighted mode) were utilized for MR analysis, with the inverse variance weighted (IVW) method being identified as the most important [15]. When the *P*_IVW_ < 0.05, as long as the beta of other methods were in the same direction as IVW method, a causal relationship between the exposure factor and the outcome factor was existing [16]. To ensure the reliability of IVW method, heterogeneity was evaluated. We calculated the index of inconsistency (*I*^*2*^) and *P*-value by Cochran’s *Q* test. When *P* < 0.05, heterogeneity was considered and random-effects IVW was used. When *P* > 0.05, no heterogeneity was considered and fixed effect IVW was used [17].

### Horizontal Pleiotropy and Sensitivity Analysis

Confounders were the exogenous factors that were related to both exposure and outcome factors in a study. If confounders were not identified or eliminated, the true relationship between exposure factor and outcome factor was hidden or exaggerated [18]. In this study, horizontal pleiotropy was calculated by the intercept of MR regression. When the intercept of MR regression was near to “0”, horizontal pleiotropy was ignored. And the results of the IVW were not affected by confounders [19]. The purpose of sensitivity analysis was to evaluate the reliability of MR. We conducted sensitivity analysis using the “leave-one-out” method. By eliminating each SNP one by one, the meta effects of the remaining SNPs were calculated to determine whether the SNP significantly altered the results. When all SNPs were on the same side of 0, the result of MR was robust.

### Mediation Analysis of BMD Levels in Different Ages

BMD levels and age have been previously confirmed as important risk factors for osteoporosis. Bone mass growth goes through a steady increase in childhood and a rapid increase in adolescence, reaching its peak and remaining stable around age 30 years [20]. Then it gradually decreases around age 50 years, with a significant decline in postmenopausal women [21]. Dual energy X-ray absorptiometry (DXA) is considered the gold standard for detecting BMD levels. The International Society for Clinical Densitometry (ISCD) recommends the lumbar spine (LS), femoral neck (FN), and total hip as preferred measurement sites for individuals over 50 years old. For children and adolescents, the recommended sites for measurement include the total body (excluding the head) and LS (http://www.iscd.org/official-positions). Therefore, on the relationship between PCSK9 inhibitors and osteoporosis, we further explored whether BMD levels with different ages had a mediation effect on osteoporosis. We selected the largest dataset of BMD levels with different ages as the mediator [22]. The database included a total of 66,628 individuals, and was divided into five stages: 0–15 years old, 15–30 years old, 30–45 years old, 45–60 years old and > 60 years old. BMD levels were measured by DXA. Total body measurements were used in children. The LS and FN were used in adults.

To ensure the reliability of the odds ratio (OR) value, SNPs significantly associated with BMD levels were used as IVs to estimate the causal effect on osteoporosis. Based on definition of the dominant model and literature report, we categorized genotypes with mutant bases as the exposed group and wild homozygotes as the non-exposed group. Here we completed the conversion of BMD levels from continuous variables into binary categorical variables [22]. Then we conducted a “two sample MR” analysis on PCSK9 inhibitors, BMD level and osteoporosis, respectively. We calculated the mediation effect and direct effect. A *P*_IVW_ < 0.05 was the prerequisite for mediation analysis, and any MR analysis that did not meet the requirements would not be subjected.

### Mediation Analysis of Serum T25(OH)D Levels

The serum T25(OH)D level is an important laboratory test for evaluating vitamin D status and its significance in relation to osteoporosis was investigated in this study. A dataset from the GWAS catalog, specifically the largest dataset (ebi-a-GCST90000618) available, was selected as the mediation factor [23]. The participants were from 2006 to 2013 and ranged in age from 40 to 60. Serum T25(OH)D levels were measured using chemiluminescence immunoassay (CLIA). Data falling outside the validation range of 10–375 nmol/L were excluded. Finally, 417,580 individuals of European ancestry were obtained. Based on definition of the dominant model and literature report, we categorized genotypes with mutant bases as the exposed group and wild homozygotes as the non-exposed group [23]. We completed the conversion of serum T25(OH)D levels from continuous variables into binary categorical variables. A “two sample MR” analysis was conducted on PCSK9 inhibitors, serum T25(OH)D levels, and osteoporosis, respectively. We calculated the mediation effect using the same method as the BMD level. A *P*_IVW_ < 0.05 was the prerequisite for mediation analysis.

### Mediation Analysis of Calcium Supplements

The effect of calcium supplements on osteoporosis is being investigated. A balanced diet is important for bone health [24]. It was suggested that calcium and vitamin D supplements may reduce fracture risk modestly [25]. Therefore, we performed a mediation analysis on calcium supplements as a possible mediation factor in this study. We selected a dataset of calcium supplements from the GWAS catalog. The dataset included 336,314 participants (case = 22,047, control = 314,267). We conducted a “two sample MR” analysis on PCSK9 inhibitors, calcium supplements and osteoporosis, respectively. A *P*_IVW_ < 0.05 was the prerequisite for mediation analysis.

## Results

### Causal Relationship between IVs and Osteoporosis

We first verified the causal relationship between PCSK9 inhibitors and CHD, as a positive control in this study. The results showed that PCSK9 inhibitors had a significant effect on CHD. This meant that PCSK9 inhibitors reduced the risk of CHD (Beta > 0, *P* < 0.05). The exposure factor selected based on PCSK9 inhibitors were effective (Supplementary Table 2). Through a “two sample MR” analysis on PCSK9 inhibitors and osteoporosis, 3 palindromic SNPs including rs12067569, rs11591147 and rs11583974 of the 13 IVs were removed. Finally, a total of 10 independent SNPs were included. We found the risk of osteoporosis was reduced by 0.6% in those who used PCSK9 inhibitors compared with non-users (OR: 0.994,* P*_IVW_ < 0.001). However, the direct correlation was weak [95% confidence interval (*CI*): 0.991–0.998]. Results of different MR methods were calculated (Table 1). Weighted median (*P* = 0.005) and weighted mode (*P* = 0.044) methods indicated a causal relationship was existing between PCSK9 inhibitors and osteoporosis. MR Egger method indicated no causal relationship was existing (*P* > 0.05). Therefore, we explored whether PCSK9 inhibitors were correlated with important factors related to osteoporosis.

**Table 1:** The causality of PCSK9 inhibitors and osteoporosis (outcome)

Exposure	MR methods	NSNP	Beta	OR (95%*CI*)	*P value*
PCSK9 inhibitors	MR Egger	10	− 0.002	0.998 (0.985–1.011)	0.769
	Weighted Median	10	− 0.006	0.994 (0.989–0.998)	0.005*
	IVW	10	− 0.006	0.994 (0.991–0.998)	< 0.001*
	Weighted mode	10	− 0.006	0.994 (0.989–0.999)	0.044*

*PCSK9* proprotein convertase subtilisin/kexin type 9; *MR* mendelian randomization; *NSNP* number of single nucleotide polymorphism; Beta, effect sizes for each SNP; *OR* odds ratio; *CI* confidence interval; *IVW* inverse variance weighted.

*P < 0.05. Taking the OR value calculated by IVW as an example, the risk of osteoporosis was reduced by 0.6% in those who used PCSK9 inhibitors compared with non-users. Beta values were used to evaluate the extent of the risk. In this study, the direct effect of PCSK9 inhibitors on the risk of osteoporosis was weak

### Heterogeneity, Horizontal Pleiotropy and Sensitivity Analysis

In this study, *I*^*2*^, Q statistic and *P* value were calculated using IVW and MR Egger methods. And the fixed-effect IVW method was adopted (Supplementary Table 3). Both IVW and MR Egger methods showed no heterogeneity (*P* > 0.05). To assess horizontal pleiotropy, the MR Egger method was used, which indicated no horizontal pleiotropy (*P* > 0.05). This suggested that osteoporosis was influenced by PCSK9 inhibitors (Supplementary Table 4). A sensitivity analysis was carried out (Supplementary Table 5). Beta values of all SNPs were less than 0 and the *P*_*IVW*_ was less than 0.05, indicating that no SNPs significantly impacted the MR results.

### Mediation Effect of BMD Level in Different Ages

**Fig. 2:**
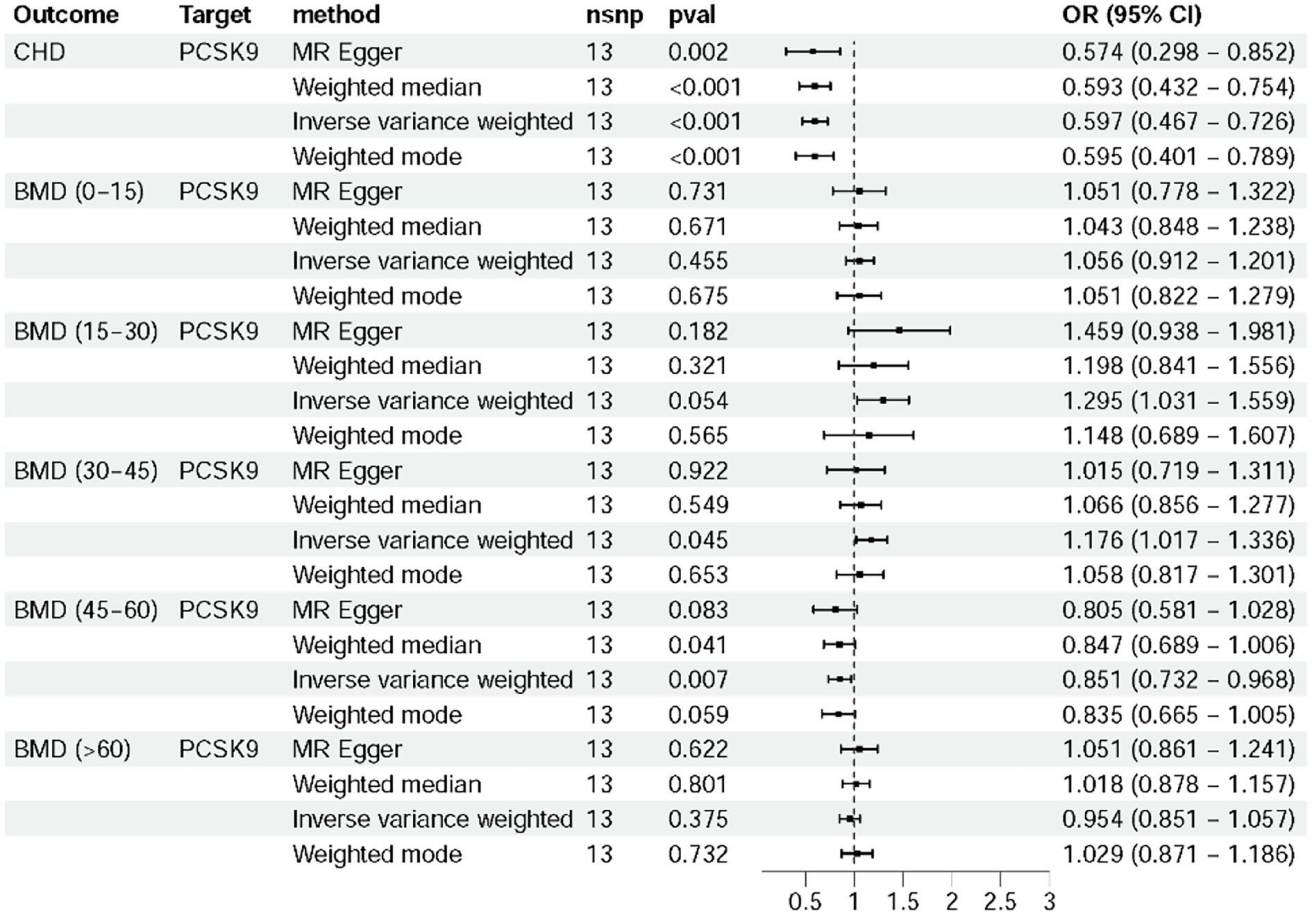
Forest plot to visualize the LDL-C level based on PCSK9 inhibitor and BMD in different ages. CHD was used as a positive control. When the *P*_*IVW*_ ≤ 0.05, PCSK9 inhibitors had a causal relationship with BMD of different ages. In people aged 30–45 years, the risk of BMD reduction was 1.176 times higher among PCSK9 inhibitor users compared to non-users. Conversely, people aged 45–60 years who used PCSK9 inhibitors had a 14.9% lower risk of BMD reduction compared to non-users. OR, odds ratio; *CI*, confidence interval; CHD, coronary heart disease; PCSK9, proprotein convertase subtilisin/kexin type 9; BMD, bone mineral density

After identifying the impact of PCSK9 inhibitors on osteoporosis, we investigated the relationship between PCSK9 inhibitors and BMD levels in different age groups. The findings revealed that in patients aged 30–45 years, the risk of low BMD was 1.176 times higher among PCSK9 inhibitor users compared to non-users (*P* = 0.045, OR: 1.176, 95%*CI*: 1.017–1.336). Conversely, people aged 45–60 years who used PCSK9 inhibitors had a 14.9% lower risk of low BMD compared to non-users (*P* = 0.007, OR: 0.851, 95%*CI*: 0.732–0.968). No causal relationship between PCSK9 inhibitors and BMD levels were observed in other age groups (Fig. 2). In the 30–45 years age group, the mediation effect and direct effect showed inconsistency (mediation effect = 0.0023, direct effect = -0.0083), with no significant effect size calculated [26]. However, in the 45–60 years age group, the mediation effect and direct effect were consistent (mediation effect = -0.0026, direct effect = -0.0034). Mediation analysis indicated that 43.33% of the effect of PCSK9 inhibitors on osteoporosis was through BMD levels, while 56.67% was a direct effect (Table 2).

**Table 2:** Mediation analysis of BMD in different ages

**Exposure**	**Outcome**	**Beta**	**Mediation effect/ direct effect**	Effect size*	***P value***
Total body BMD (age 30–45 years)					
PCSK9 inhibitor	BMD	− 0.163^b1^	−	−	0.046
BMD	osteoporosis	− 0.014^b2^	−	−	2.162×10^−12^
PCSK9 inhibitor	osteoporosis	− 0.006^b^	0.0023/ − 0.0083	−	0.007
Total body BMD (age 45–60 years)					
PCSK9 inhibitor	BMD	0.162^b3^	−	−	0.007
BMD	osteoporosis	− 0.016^b4^	−	−	1.286×10^−43^
PCSK9 inhibitor	osteoporosis	− 0.006^b^	− 0.0026/ − 0.0034	43.33%	0.007

*BMD* bone mineral density; Beta, effect sizes for each SNP; *PCSK9* proprotein convertase subtilisin/kexin type 9. Mediation effects (age 30–45 years) = b1 × b2, direct effect (age 30–45 years) = b-b1 × b2; mediation effects (age 45–60 years) = b3 × b4, direct effect (age 45–60 years) = b-b3 × b4. Effect size = b3 × b4/b

### Mediation Effect of Serum T25(OH)D Levels

**Fig. 3:**
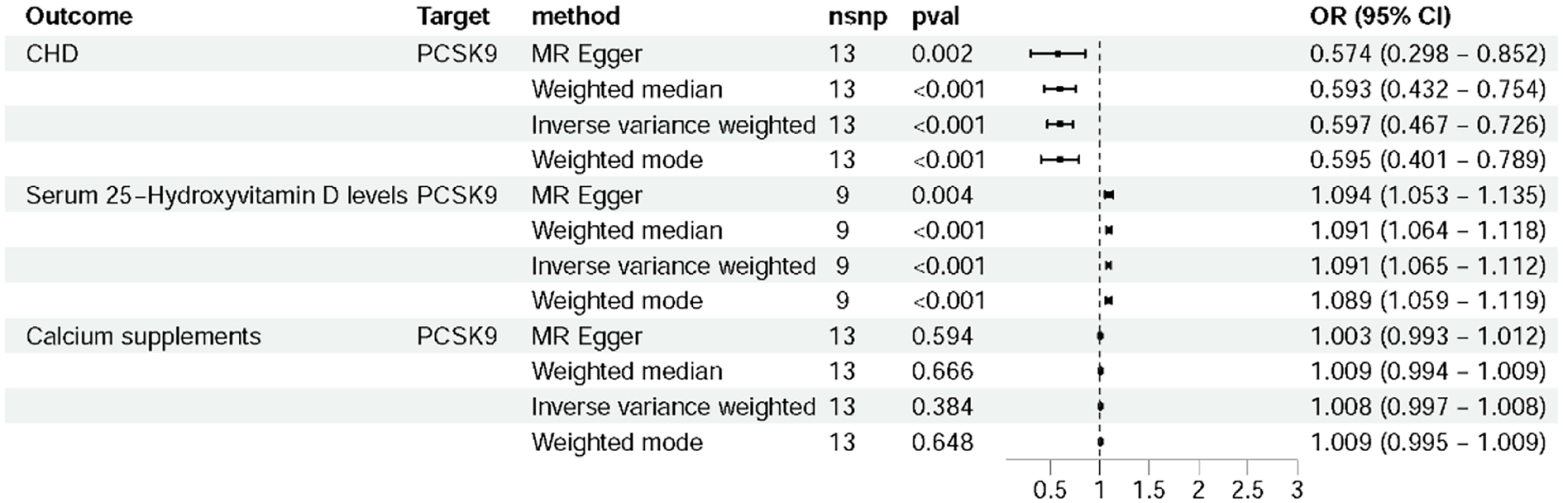
Forest plot to visualize the LDL-C level based on PCSK9 inhibitors with serum 25-hydroxyvitamin D levels and calcium supplements. When the *P*_*IVW*_ ≤ 0.05, the risk of high serum T25(OH)D levels were 1.091 times higher among PCSK9 inhibitor users compared to non-users. OR, odds ratio; *CI*, confidence interval; CHD, coronary heart disease; PCSK9, proprotein convertase subtilisin/kexin type 9

Serum T25(OH)D levels served as a common laboratory indicator for osteoporosis. In this study, we investigated the correlation between PCSK9 inhibitors and serum T25(OH)D levels (Table 3). The IVW analysis revealed that the risk of high serum T25(OH)D levels were 1.091 times among PCSK9 inhibitor users compared to non-users (OR: 1.091, 95%*CI*: 1.065–1.112) (Fig. 3). However, the MR results did not show statistical significance in the relationship between serum T25(OH)D levels and osteoporosis (*P* = 0.239) (Table 3). Despite the observed increase in serum T25(OH)D levels following PCSK9 inhibitor administration, this study did not establish a causal relationship between serum T25(OH)D levels and osteoporosis. Consequently, the mediation effect was not calculated.

**Table 3:** Mediation analysis of serum total 25-hydroxyvitamin D levels

Exposure	Outcome	Beta	Mediation effect	*P value*
PCSK9 inhibitor	T25(OH)D	− 0.087^b1^	−	1.784×10^−11^*
T25(OH)D	osteoporosis	0.001^b2^	−	0.239
PCSK9 inhibitor	osteoporosis	− 0.006^b^	− 8.70×10^−5^	0.007*

*NSNP* number of single nucleotide polymorphism; Beta, effect sizes for each SNP; *PCSK9* proprotein convertase subtilisin/kexin type 9; *T25(OH)D* total 25-hydroxyvitamin D; *OP* osteoporosis.

*P < 0.05. Beta values represented standardized regression coefficients used to evaluate the influence of exposure factors on the occurrence of outcome factors

### Mediation Effect of Calcium Supplements

We investigated the correlation between PCSK9 inhibitors and calcium supplements. MR results showed that there was no causal relationship between PCSK9 inhibitors and calcium supplements (Fig. 3). Therefore, no mediation analysis was conducted.

## Discussion

By combining drug-targeted MR with mediation analysis, we explored the relationship between PCSK9 inhibitors and the risk of osteoporosis. IVW results indicated that while PCSK9 inhibitors helped decrease the occurrence of osteoporosis, the direct correlation was found to be weak. Furthermore, we observed varying effects of PCSK9 inhibitors on BMD levels based on patient age. Specifically, in patients aged 30–45 years, PCSK9 inhibitors were associated with increased risk of low BMD levels, whereas in patients aged 45–60 years, they were protective. Mediation analysis revealed that BMD levels partially mediated the risk of osteoporosis in the 45–60 age group. The indirect effect of PCSK9 inhibitors on osteoporosis through BMD accounted for 43.33%, while the direct effect accounted for 56.67%. These findings have significant implications for guiding clinical drug use.

As a key factor regulating the expression of LDL-C, a variety of antibody drugs targeting PCSK9 have been developed and applied to clinical, such as alirocumab and evolocumab [27]. The drugs significantly reduced LDL-C levels and played an important role in the treatment of cardiovascular with poor response to statin drugs [28]. Recent research on LDL-C has expanded beyond CHD to include a broader range of conditions. A study of 4,909 adults aged 20–59 years indicated a negative association between LDL-C and lumbar bone density, suggesting a potential value for LDL-C in early osteoporosis detection [4]. However, this study did not stratify patients by age, leaving uncertainty regarding the clinical implications of LDL-C across all age groups. Clinical trials have demonstrated the efficacy and safety of PCSK9 inhibitors in stroke prevention [29]. Additionally, research on PCSK9 inhibitors and malignant tumors has shown varying associations, with negative correlations observed with breast and lung cancer, but positive correlations with gastric, liver, oral, pharyngeal, and cervical cancers [30]. To further explore the relationship between PCSK9 inhibitors and osteoporosis, we conducted drug-targeted MR analysis. Combining this approach with mediation analysis help we enhance our understanding of the effects of PCSK9 inhibitors on osteoporosis.

The LDL-C dataset for this study included individuals from various ethnic backgrounds. Previous studies have indicated that the impact of a particular exposure on a disease may vary across different ethnic groups [31, 32]. To validate the reliability of this exposure factor, a mixed dataset of CHD was chosen as a positive control. The findings demonstrated a causal relationship between LDL-C and CHD among patients from different ethnicities. Lowering LDL-C levels was found to decrease the risk of CHD, a result that has been consistently supported by studies involving different populations [33–35]. Subsequently, we tested horizontal pleiotropy and did not find the presence. This indicated that the study was not influenced by confounding variables. Furthermore, numerous lipid-lowering treatments utilizing PCSK9 inhibitors have proven to be effective across various ethnic groups [36–38]. As a result, it was believed that the ethnic diversity in this study did not impact the relationship between the exposure and outcome factors.

IVW method is a commonly used method in MR analysis. When using multiple algorithms such as MR Egger, weighted median, IVW, and weighted mode for MR analysis, it is essential to satisfy at least two key criteria. Firstly, the IVW results should demonstrate significance. Secondly, the beta coefficients of different algorithms should exhibit consistency [39]. In this study, our findings indicated that the IVW result was significant, and the beta coefficients of other MR algorithms were in alignment. Subsequently, in a follow-up investigation, we adhered to the same criteria and observed that combining the IVW method with beta coefficients was beneficial in uncovering and exploring the relationship between PCSK9 inhibitors and osteoporosis.

In this study, it was observed that PCSK9 inhibitors had differing effects on BMD levels in patients aged 30–45 years compared to those aged 45–60 years. Specifically, PCSK9 inhibitors promoted the reduction of BMD levels in the younger age group, while protecting BMD levels in the older age group. This age-dependent phenomenon has significant implications for clinical practice. Previous studies have highlighted the importance of age as a key factor for osteoporosis [40, 41]. Inflammation and senescence associated phenotype (SASP) have been identified as key contributors to osteoporosis pathogenesis [42]. Serum tumor necrosis factor α (TNF-α) and interleukin-20 (IL-20) were shown to stimulate the expression of receptor activator of nuclear factor kappa B (RANK) in osteoclast progenitors and RANK ligand (RANKL) in osteoblasts, leading to increased osteoclast formation. The increase of osteoclast production accelerated the thinning of bone trabeculae and calcium loss, which eventually led to the occurrence of osteoporosis [43]. The findings of this study in patients aged 45–60 years align with previous research. In addition, mediation analysis showed that PCSK9 inhibitors prevented osteoporosis by both direct effect and mediation effect. The direct effect on osteoporosis accounted for 43.33%, and the indirect effect accounted for 56.67%. It has not been reported in previous studies. This insight is not only the verification of the two-sample MR analysis, but also the extension of the MR analysis.

The results in this study suggested that PCSK9 inhibitors could reduce BMD levels in patients aged 30–45 years, a phenomenon not previously reported. We analyzed the problem in two ways. Initially, it was only analyzed from the MR’s results. Despite the IVW method indicating a notable discrepancy between PCSK9 inhibitors and BMD levels, the *P* value was close to 0.05, which was different from the significant *P* value in the 45–60 years group. It was noted that a smaller *P* value (e.g., *P* < 0.001) often provided more robust evidence of effectiveness compared to *P* < 0.05 [44]. For instance, the clinical approval of LCZ696 over enalapril for heart failure was based on a smaller *P* value (*P* < 0.00001) [19]. Conversely, in the NXY-059 trial for acute ischemic stroke, the *P* value of primary outcome measure was 0.038. Although below 0.05, this value was not enough to justify the efficacy of NXY-059. No significant effect of NXY-059 was found in a larger clinical trial (*P* = 0.33) [44]. Consequently, the evidence from clinical trials suggested that NXY-059 was likely ineffective in treating acute ischemic stroke. These studies suggested that positive results with a *P* value close to 0.05 should be interpreted with caution and proved by extensive clinical validation.

The second aspect was the magnitude of treatment efficacy. It was necessary to assess the level of uncertainty through the 95% *CI*. In a clinical trial involving simvastatin for acute coronary syndrome, the hazard ratio for composite primary outcomes in patients using ezetimibe was 0.94 (95% *CI*: 0.89–0.98, *P* = 0.016). Although the results were described as positive, the benefits of ezetimibe were insufficient [45]. Additionally, the OR values of other MR methods were close to 1, suggesting a significant level of uncertainty based on statistical results. The practical clinical relevance of these findings would need to be confirmed through studies involving a larger population.

We also explored the mediation effects of serum T25(OH)D levels and calcium supplements. Despite not identifying a mediation effect between serum T25(OH)D levels and calcium supplements, the MR analysis revealed that PCSK9 inhibitors were associated with an increase in serum T25(OH)D levels. Total 25(OH)D level was utilized as an indicator of vitamin D status, with T25(OH)D binding to vitamin D-binding protein (DBP) and albumin in peripheral blood circulation. However, only free 25(OH)D index was significantly associated with BMD [46]. We did not find a causal relationship between serum T25(OH)D levels and osteoporosis, consistent with prior research [47, 48]. Notably, vitamin D deficiency was linked to increased risk of all-cause mortality in patients with chronic kidney disease [49]. And T25(OH)D deficiency was an independent prognostic factor in patients with B chronic lymphocytic leukemia or non-Hodgkin lymphoma [50]. Furthermore, serum T25(OH)D levels in children were negatively correlated with asthma [51]. These findings underscored the importance of serum T25(OH)D levels for improving disease prognosis. In this study, although we did not find a mediation effect of serum T25(OH)D levels, we did find a causal relationship between PCSK9 inhibitors and serum T25(OH)D levels. Whether PCSK9 inhibitors could elevate serum T25(OH)D levels and improve osteoporosis, was of big value for further clarifying.

This study also has some limitations. At first, the exposure factor included datasets of different ethnicities. Whether the heterogeneity between ethnicities would lead to the different effects on the same outcome still needed to be verified separately in different ethnicities. Second, we only stratified ages in this study and did not stratify gender and were unable to retrieve dataset of serum free 25(OH)D levels in public databases, and were unable to perform mediation analysis. In addition, since the datasets on T25(OH)D levels in public databases were not stratified by age or gender, we will search databases to conduct more detailed research in the future. At the same time, we will also explore the relationship between different types of PCSK9 inhibitors and serum LDL-C levels, serum T25(OH)D levels, calcium supplements, and osteoporosis. These are the areas that we need to make up in the future.

## Conclusion

A causal relationship was found between LDL-C levels based on PCSK9 inhibitors and osteoporosis. The impact of PCSK9 inhibitors on BMD levels varied across different age groups. In individuals aged 45–60 years, PCSK9 inhibitors were observed to prevent osteoporosis by increasing BMD levels. Additionally, PCSK9 inhibitors were found to elevate serum T25(OH)D levels, providing valuable insights for clinicians.

### Supplementary Information

Below is the link to the electronic supplementary material.

Supplementary file1 (DOCX 19 KB)

Supplementary file2 (DOCX 25 KB)

Supplementary file3 (DOCX 25 KB)

Supplementary file4 (DOCX 34 KB)

## Data Availability

All data were available at the GWAS Catalog and MR Base database. The positive control was CHD (ieu-a-7). The exposure was LDL-C (ieu-a-300). The outcome was osteoporosis (ukb-b-12141).
