# Supplementary material for: The Causal Relationship between PCSK9 Inhibitors and Osteoporosis Based on Drug-Targeted Mendelian Combined Mediation Analysis
Source: Calcif Tissue Int. 2024 May 24;115(1):53–62. doi: 10.1007/s00223-024-01228-x (PMC11153280; doi:10.1007/s00223-024-01228-x)
Supplement: Supplementary file 2 — Supplementary file2 (DOCX 25 KB) [file 223_2024_1228_MOESM2_ESM.docx]

**Supplementary Table 1** Characteristics of instrumental variables

| **SNP (IVs*****)** | **Chr** | **Number of SNPs** | **Beta** | **Se** | ***P*** |
| --- | --- | --- | --- | --- | --- |
| rs2495495 | 1 | 162403 | -0.034 | 0.0059 | 3.520🞨10^-8^ |
| rs2495477 | 1 | 80151 | -0.064 | 0.0054 | 7.285🞨10^-30^ |
| rs2479409 | 1 | 172970 | -0.064 | 0.0041 | 2.515🞨10^-50^ |
| rs12067569 | 1 | 164264 | -0.089 | 0.0100 | 1.966🞨10^-17^ |
| rs10493176 | 1 | 86056 | -0.078 | 0.0102 | 2.535🞨10^-14^ |
| rs11591147 | 1 | 77417 | -0.497 | 0.0180 | 8.570🞨10^-143^ |
| rs4927193 | 1 | 173009 | -0.035 | 0.0056 | 4.269🞨10^-11^ |
| rs11583974 | 1 | 99955 | 0.065 | 0.0117 | 3.951🞨10^-19^ |
| rs2479394 | 1 | 172953 | -0.039 | 0.0041 | 1.584🞨10^-19^ |
| rs11206510 | 1 | 172812 | -0.083 | 0.0050 | 2.380🞨10^-53^ |
| rs572512 | 1 | 150564 | 0.048 | 0.0047 | 5.309🞨10^-26^ |
| rs11206514 | 1 | 172996 | 0.051 | 0.0041 | 9.949🞨10^-33^ |
| rs585131 | 1 | 167769 | 0.064 | 0.0050 | 2.699🞨10^-35^ |

SNP, single nucleotide polymorphism; Chr, chromosome; IVs, instrumental variables. IVs*: instrumental variables (SNPs of low density lipoprotein cholesterol that were located within ± 100 clumping distance of proprotein convertase subtilisin/kexin type 9); Beta; effect sizes for each SNP. Beta<0 meant the exposure played a reverse role in the outcome, beta>0 meant exposure played a positive role in the outcome.

**Supplementary Table 2** The causality of PCSK9 inhibitors and coronary heart disease (outcome)

| **Exposure** | **MR methods** | **NSNP** | **Beta** | **OR (95%*CI*)** | ***P*** |
| --- | --- | --- | --- | --- | --- |
| PCSK9 inhibitors | MR Egger | 13 | 0.553 | 0.574 (0.298-0.851) | 2.393🞨10^-3^ |
|  | Weighted Median | 13 | 0.522 | 0.593 (0.432-0.754) | 1.792🞨10^-10^ |
|  | IVW | 13 | 0.516 | 0.596 (0.467-0.726) | 6.579🞨10^-15^ |
|  | Weighted mode | 13 | 0.519 | 0.595 (0.401-0.789) | 2.493🞨10^-4^ |

PCSK9, proprotein convertase subtilisin/kexin type 9; MR, mendelian randomization; NSNP, number of single nucleotide polymorphism; IVW, inverse variance weighted; Beta, effect sizes for each SNP; OR, odds ratio; *CI*, confidence interval. OR<1 meant protective effect, OR>1 meant promoting effect.

**Supplementary Table 3** Heterogeneity of instrumental variables

| **Instrumental variables** | **MR methods** | **Cochran *Q* statistic** | ***I****^2^* | **Degree of freedom** | ***P*** |
| --- | --- | --- | --- | --- | --- |
| PCSK9 inhibitors | MR Egger | 6.560 | 0.762 | 8 | 0.585 |
|  | IVW | 6.885 | 0.878 | 9 | 0.649 |

PCSK9, proprotein convertase subtilisin/kexin type 9; MR, Mendelian randomization; *I^2^*, index of inconsistency; IVW, inverse variance weighted.

**Supplementary Table 4** Horizontal pleiotropy of instrumental variables

| **Instrumental variables** | **MR Egger** | | **MR-PRESSO** | |
| --- | --- | --- | --- | --- |
|  | **intercept** | ***P value*** | **T-stat** | ***P value*** |
| PCSK9 inhibitors | -0.0002 | 0.584 | -3.834 | 0.720 |

MR, Mendelian randomization; PCSK9, proprotein convertase subtilisin/kexin type 9.

**Supplementary Table 5** The sensitivity analysis of MR

| **SNP** | **Sample size** | **Beta** | **Se** | **OR (95%*CI*)** | ***P value*** |
| --- | --- | --- | --- | --- | --- |
| rs10493176 | 462933 | -0.0061 | 0.0018 | 0.997 (0.984-1.010) | 0.0007 |
| rs11206510 | 462933 | -0.0054 | 0.0019 | 0.991 (0.984-1.000) | 0.0057 |
| rs11206514 | 462933 | -0.0062 | 0.0018 | 0.996 (0.986-1.007) | 0.0009 |
| rs2479394 | 462933 | -0.0055 | 0.0018 | 0.988 (0.973-1.003) | 0.0021 |
| rs2479409 | 462933 | -0.0057 | 0.0019 | 0.993 (0.985-1.002) | 0.0030 |
| rs2495477 | 462933 | -0.0063 | 0.0019 | 0.996 (0.988-1.005) | 0.0009 |
| rs2495495 | 462933 | -0.0054 | 0.0017 | 0.974 (0.951-0.997) | 0.0021 |
| rs4927193 | 462933 | -0.0058 | 0.0017 | 0.991 (0.971-1.012) | 0.0011 |
| rs572512 | 462933 | -0.0066 | 0.0018 | 1.002 (0.991-1.014) | 0.0003 |
| rs585131 | 462933 | -0.0056 | 0.0018 | 0.992 (0.981-1.002) | 0.0024 |
| All-IVW | 462933 | -0.0058 | 0.0017 | 0.994 (0.991-0.998) | 0.0008 |
| ALL-MR Egger | 462933 | -0.0021 | 0.0068 | 0.998 (0.984-1.011) | 0.7693 |

MR, mendelian randomization; SNP, single nucleotide polymorphism; Beta, effect sizes for each SNP; OR, odds ratio; *CI*, confidence interval.
