# Supplementary material for: The Causal Relationship between PCSK9 Inhibitors and Osteoporosis Based on Drug-Targeted Mendelian Combined Mediation Analysis
Source: Calcif Tissue Int. 2024 May 24;115(1):53–62. doi: 10.1007/s00223-024-01228-x (PMC11153280; doi:10.1007/s00223-024-01228-x)
Supplement: Supplementary file 1 — Supplementary file1 (DOCX 19 KB) [file 223_2024_1228_MOESM1_ESM.docx]

**Dear Reviewer，**

Thank you for your letter for the comments concerning our manuscript entitled “**The causal** **relationship between PCSK9 inhibitors and osteoporosis based on drug targeting Mendelian combined mediation analysis**” (ID: CTIN-D-23-00467R3). We have studied comments carefully and have made correction which we hope meet with approval. We highlight the changes within the document by making in red in revised paper. The main corrections in the paper and the responds to the reviewer and editor comments are as flowing:

**Response to reviewer** #1**：**

**Q1、**The statement "the risk of osteoporosis was reduced by 0.6% in those who used PCSK9 inhibitors compared with non-users (OR: 1.006, 95%CI: 1.002-1.009, *P*<0.001)" does not look correct as an odds ratio of 1.006 corresponds to an increase in risk of 0.6%. Please check this statement. Should the correct estimates be (OR: 0.994, 95%CI: 0.991-0.998)?

**A1:** Thank you very much for pointing out our problems. We carefully checked the data in **Table 1** and found that we made mistakes in the statement of abstract. We revised the wrong description (L23-L25) and marked it with red. Thanks again for pointing out our mistake.

**Q2、**The statement "serum T25(OH)D levels were 1.091 times higher among PCSK9 inhibitor users compared to non-users (OR: 1.091, 95%CI: 1.065-1.112, *P*<0.001)" does not look correct as an odds ratio greater than one must refer to increased risk of some outcome and not the amount that the levels of a continuous outcome increase. Therefore, I expect that the statement should read as either "the risk of high serum T25(OH)D levels were 1.091 times higher among PCSK9 inhibitor users compared to non-users (OR: 1.091, 95%CI: 1.065-1.112, *P*<0.001)" OR "the risk of low serum T25(OH)D levels were 1.091 times higher among PCSK9 inhibitor users compared to non-users (OR: 1.091, 95%CI: 1.065-1.112, *P*<0.001)". Please clarify these statements which describe the association between use of PCSK9 inhibitors and serum T25(OH)D levels in all the parts of the document where they are stated.

**A2:** Thanks to your guidance. We have revised the statements which describe the association between use of PCSK9 inhibitors and serum T25(OH)D levels in figure (L29-L31), abstract (L31-L32), manuscript (L212-L213). We marked these with red. I hoping to get your approval.

I would like to express my thanks to the reviewer for the guidance. In order to help us improve the quality of manuscript, the reviewer gave us patient guidance again and again. Under your guidance, this manuscript has been greatly improved compared with the original submission, which is inseparable from the hard work of the reviewer. We appreciate for reviewer’ warm work. Once again, thank you very much for your comments and suggestions.

Yours sincerely,

Corresponding author: Naidan Zhang

E-mail: [znd1984@126.com](mailto:znd1984@126.com)
